# Contribution of multimodal ultrasound in evaluating the efficacy of lumbricus protein fast dissolving tablets against atherosclerotic plaques in ApoE^(−/−)^ mice

**DOI:** 10.3389/fphar.2025.1551833

**Published:** 2025-05-30

**Authors:** Zhihao Lu, Ning Wang, Quanlin Yu, Jing Feng, Jianteng Dong, Xu Zhao, Chengke Cai, Jian Li, Liqun Zhong

**Affiliations:** ^1^ School of Traditional Chinese Medicine, Beijing University of Chinese Medicine, Beijing, China; ^2^ Dongzhimen Hospital, Beijing University of Chinese Medicine, Beijing, China; ^3^ School of Chinese Materia Medica, Beijing University of Chinese Medicine, Beijing, China

**Keywords:** 3R principles, lumbricus protein orally disintegrating tablets, atherosclerotic plaques, ApoE−/−mice, multimodal ultrasound

## Abstract

**Introduction:**

Atherosclerosis (AS), a prevalent cardiovascular condition characterized by cholesterol accumulation, inflammation, and fibrous tissue proliferation within arterial walls, remains a major global health challenge. Traditional Chinese Medicine (TCM) identifies earthworm (Lumbricus) as an effective treatment for blood stasis syndromes. Some studies have identified lumbricus-derived extracts as being rich in collagenase and fibrinolytic enzymes, which has significant effects in dissolving blood clots, improving circulation, and preventing thrombosis. Therefore, in the present study, a novel formulation, fast dissolving tablets of lumbricus protein (abbreviated as: LP-FDT), was developed as part of upcoming new drug research and development, and we used multi-modal ultrasonic technique combined with routine biochemical and histopathological analysis methods to evaluate its efficacy on AS. By leveraging the advantages of LP-FDT and adhering to the principle of the 3Rs (replacement, reduction, refinement), this research offers a novel and sustainable therapeutic strategy for managing AS. The findings from this study are expected to provide valuable insights into the development of patient-friendly treatments for AS, bridging the gap between traditional therapies and modern pharmaceutical innovations.

**Methods:**

Animal model was established by ApoE-/- feeding high-fat diet for 8 weeks. Multimodal ultrasound, along with histopathological and biochemical analyses, was employed to assess the therapeutic effects.

**Results:**

LP-FDT significantly reduced arterial plaque size and inflammation while enhancing collagen remodeling within the plaques. Although no substantial impact on serum lipid profiles was observed, LP-FDT significantly downregulated MMP-2 and MMP-9 expression, suggesting a novel immunomodulatory mechanism in extracellular matrix degradation and plaque stabilization.

**Discussion:**

In this study, we confirmed the efficacy of LP-FDT for AS by multimodal ultrasound, along with histopathological and biochemical analyses. At the same time, by adhering to the principles of the 3Rs (replacement, reduction, refinement), the study minimized animal use and suffering while maximizing experimental reliability. The findings of this study indicate that LP-FDT, an innovative formulation combining TCM principles with modern pharmaceutical technologies, holds significant promise in the prevention and treatment of AS, providing a new pathway for the integration of traditional and contemporary approaches to cardiovascular health. Further investigation into its molecular mechanisms is warranted.

## 1 Introduction

Atherosclerosis (AS), a multifaceted pathological process affecting the arteries, has become a global public health issue due to its severe threat to human health. The WHO’s 2023 report highlights the preeminence of cardiovascular disease as the leading cause of both incidence and mortality among non-communicable diseases (World Health Organization, 2023). AS is characterized by a range of pathological changes within the arterial intima, including cholesterol lipid accumulation, inflammation, fibrous tissue proliferation, and calcification (Halasz and Piepoli, 2021; Xiang et al., 2022; Libby, 2021a; Wolf and Ley, 2019; Badimon and Vilahur, 2014; Bennett et al., 2016; Shrestha et al., 2014; Durham et al., 2018). These alterations culminate in the formation of atheromatous plaques, which progressively narrow vessel lumens, compromise elasticity, and ultimately precipitate grave cardiovascular sequelae such as myocardial thrombosis, infarction, and stroke (Badimon and Vilahur, 2014; Marston et al., 2022; Falk, 2006). The high prevalence of AS further emphasizes its status as a pressing global public health challenge that requires urgent resolution. In Traditional Chinese medicine (TCM), AS is often associated with patterns of blood stasis (*Xue Yu*), qi deficiency (*Qi Xu*), and phlegm dampness (*Tan Shi*), which disrupt the proper flow of blood in the vessels and lead to disease. Regarding this, the principle of invigorating blood and dispelling stasis (*Huoxue Huayu*) is a key strategy on the treatment of AS. Lumbricus (earthworm) is classified within the invigorating blood and dispelling stasis category in TCM and is widely used in the treatment of blood stasis conditions involving AS (Xiang et al., 2015). Some studies have identified lumbricus-derived extracts as being rich in collagenase and fibrinolytic enzymes, which has significant effects in dissolving blood clots, improving circulation, and preventing thrombosis (Nakajima et al., 1993; Cooper et al., 2004; Tjandrawinata et al., 2014). Additionally, lumbricus protein is included in the medicinal and edible products approved by the China Food and Drug Administration (CFDA). Given its safety and effectiveness, lumbricus protein has been developed into different functional foods or medicines. In the present study, a novel formulation, fast dissolving tablets of lumbricus protein (abbreviated as: LP-FDT), was developed as part of upcoming new drug research and development. The tablet form preserves the original activity of lumbricus protein while benefiting from a unique hydrophilic, loose, and porous structure. This allows for rapid disintegration and dispersion upon contact with water, enabling the active ingredients to be instantly released and absorbed through the oral mucosa for quick effect. Despite these advantages, there is a notable lack of systematic research investigating whether LP-FDT can alleviate the formation of atherosclerotic plaques, highlighting a significant gap in knowledge that urgently needs to be addressed. In the present study, we used a well-established ApoE knock out mouse model (ApoE^−/−^) fed with a high fat diet. We also used multi-modal ultrasonic technique combined with routine biochemical and histopathological analysis methods to evaluate the efficacy of each intervention before the study endpoint. By leveraging the advantages of LP-FDT and adhering to the principle of the 3Rs (replacement, reduction, refinement) (Rinwa et al., 2024; Obrecht et al., 2023), this research offers a novel and sustainable therapeutic strategy for managing AS. The findings from this study are expected to provide valuable insights into the development of patient-friendly treatments for AS, bridging the gap between traditional therapies and modern pharmaceutical innovations.

## 2 Materials and methods

### 2.1 Materials

SPF-grade APOE^−/−^ mice, 8 weeks old, male, body weight 22 ± 2 g, genetic background C57BL/6J were selected for this study. The mice were housed in the laboratory animal center of Beijing University of Traditional Medicine, animal license number SCXK (Beijing) 2021-0006. The High fat feed (Cat. H10141, formula: 77.85% maintenance diet, 22% fat, 0.15% cholesterol) was purchased from Beijing Vital River Laboratory Animal Technology Co., Ltd. The experimental drug LP-FDT was made by our lab (see supporting data 1). The following kits and reagents were used in the study: Oil Red O staining kit (G1261-2, 100 mL), Masson staining kit (Cat: G1346), TG kit (A110-1-1), TC kit (A111-1-1), HDL-C kit (A112-1-1), LDL-C kit (A113-1-1), MMP-9(Cat:10375-2-AP), MMP-2(Cat:10373-2-AP), Col1A1(Cat No.67288-1-Ig), CD68 (Anti-CD68 antibody [KP1] (ab955)) immunohistochemical kit (E2056), and Atorvastatin (H20051407). The multimodality ultrasound instrument (LOGIQ Fortis) and multimodality ultrasound probe (L6-24) were purchased from GE Healthcare (United States).

The experiment was approved by the experimental animal ethics committee of Beijing University of Chinese Medicine (ethical approval number: BUCM-2023032901-1143).

### 2.2 Methods

#### 2.2.1 Animal experiment design

**FIGURE 1:**
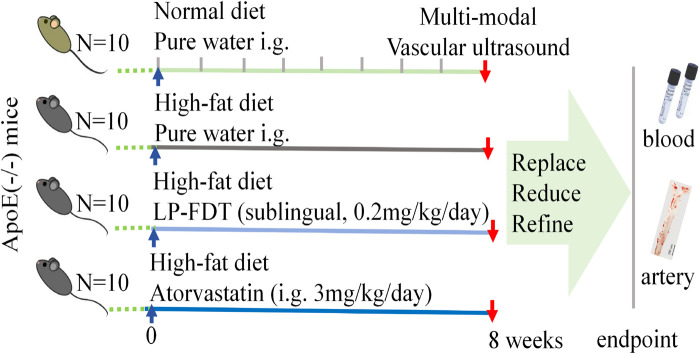
Experimental flow design.

As showed in Figure 1, after a 1-week acclimatization period, 40 APOE^−/−^ mice were randomly divided into 4 groups (10 mice/group): ⅰ. Control group: normal diet with purified water treatment (100 μL, i. g.); ⅱ. Model group: high-fat diet with purified water treatment (100 μL, i. g.); ⅲ. LP-FDT group: high-fat diet with LP-FDT treatment (0.2 mg/kg/day, sublingual administration); ⅳ. Positive drug control: high-fat diet with atorvastatin treatment (3 mg/kg/d, i. g.). The duration of intervention was 8 weeks, with all treatments starting concurrently with model induction.

#### 2.2.2 Multi-modal vascular ultrasound detection

The mice in each group were sequentially examined by ultrasound. In brief, mice were anesthetized with a small animal anesthesia machine filled with isoflurane. The hair in the neck and chest areas were thoroughly removed to fully expose the operative field. The mice were then gently placed in a supine position on a 37°C blanket to simulate their physiological body temperature environment and maintain stable vital signs. A high-precision ultrasound probe was used to scan the aortic arch area of the mice. Throughout this process, the operator strictly maintained a consistent ultrasound detection depth to ensure the accuracy and reproducibility of the acquired data, including the size and morphology of vascular plaques, carotid blood flow, and echogenicity, which were crucial for accurately locating the carotid artery.

#### 2.2.3 Serum biological examination

The serum levels of total cholesterol (TC), triglycerides (TG), low-density lipoprotein cholesterol (LDL-C), and high-density lipoprotein cholesterol (HDL-C) of each mouse were measured using the corresponding kits. The absorbance value of each well was measured with a microplate reader, with a wavelength of 600 nm for HDL-C and LDL-C detection, and 500 nm for TG and TC detection. Serum lipid levels were calculated according to the kit instructions.

#### 2.2.4 Histopathological examination

The entire aorta was excised and fixed in a 10% formalin solution, and the atherosclerotic plaques located in the aortic intima were examined by Oil Red O staining. The aortic root was harvested, fixed in a 10% formalin solution, paraffin embedded, and sectioned at 6 μm thickness. For histological analysis, Hematoxylin and Eosin (H&E) staining was performed to assess the general tissue architecture and cellular morphology. Masson’s staining was also carried out to distinguish collagen fibers and to assess the extent of extracellular matrix (ECM) remodeling within the plaques and surrounding aortic tissue. The stained sections were examined under a light microscope, and digital images were captured for further analysis of histological changes, plaque composition, and ECM extent.

#### 2.2.5 Immunohistochemistry (IHC) analysis

For IHC, tissue sections were dewaxed, rehydrated, and blocked with 5% goat serum and incubated with primary antibody targeting CD68 (1:100), Col1A1 (1:100), MMP-2 (1:500) and MMP-9 (1:500) at 4°C overnight. After 3 washes with PBS, the sections were incubated with secondary antibody at 37°C for 1 h. The DAB kit was used to visualize the positive signals in the sections. Cell nuclei were counterstained with hematoxylin solution. ImageJ was used to calculate the integrated optical density of the positive area of unit area.

#### 2.2.6 Real-time quantitative RT-PCR detection

The total RNA was extracted using Trizol regent. The extracted RNA was reverse transcribed by an RNA reverse transcription kit. The quantitative RT-PCR was configured, and the OD values were measured by a real-time PCR instrument. The data were analyzed according to 2^-△△ct^. The RT-PCR primer of MMPs were listed in the Supplementary Table S1 (Supporting data 2).

#### 2.2.7 Statistical methods

The results of multimodal ultrasound, blood lipid levels, positive expression data from aortic arch IHC staining, and 2^-△△ct^ data from PCR were statistically analyzed and plotted by Graphad Prism 9.0 software. One-way analysis of variance (one-way ANOVA) was used to compare measurement data between groups, assuming homogeneous variances, and the Least Significant Different (LSD) method was used for pairwise comparison. If variances were not uniform, the LSD method was used after data transformation. The significance level was *P* < 0.05, and data are presented as mean $\bar{x}\pm SD$ (standard deviation).

## 3 Results

### 3.1 Intravascular plaque and blood flow velocity detection

**FIGURE 2:**
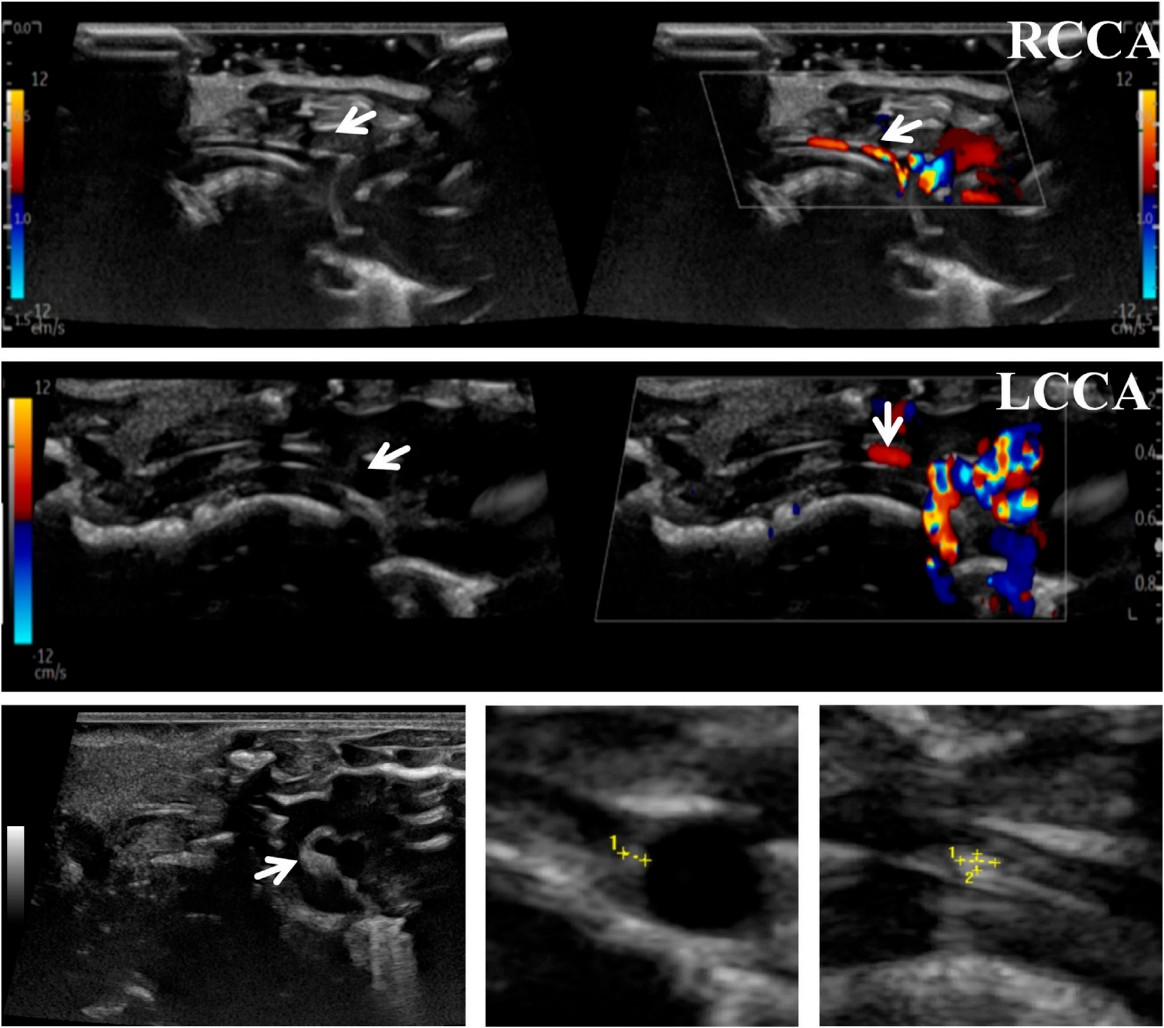
Imaging features of arterial vessels and plaques detected by ultrasound.

**FIGURE 3:**
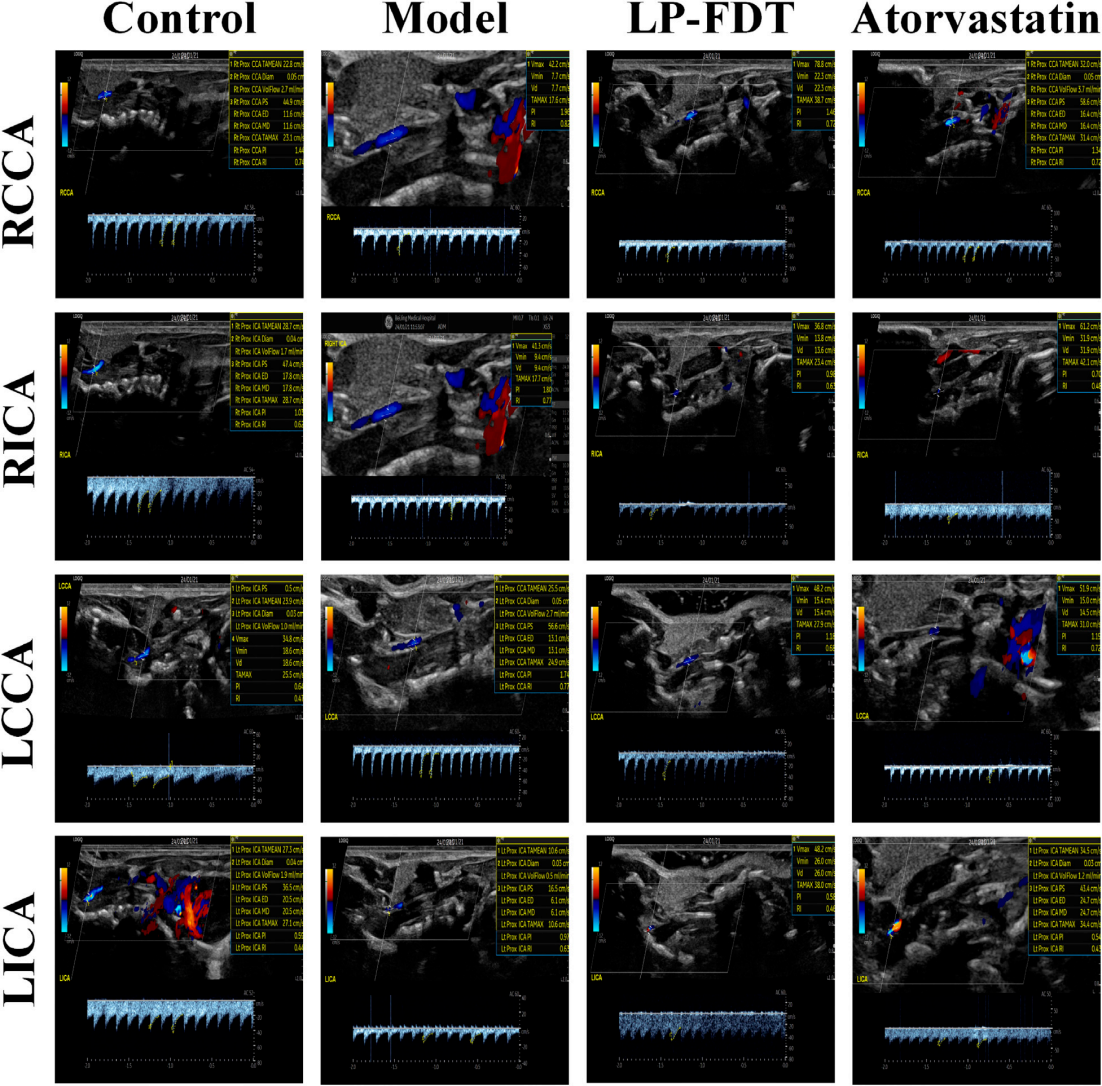
Blood flow velocity and blood flow related indexes of four carotid arteries (RCCA, RICA, LCCA, LICA) detected by ultrasound.

**FIGURE 4:**
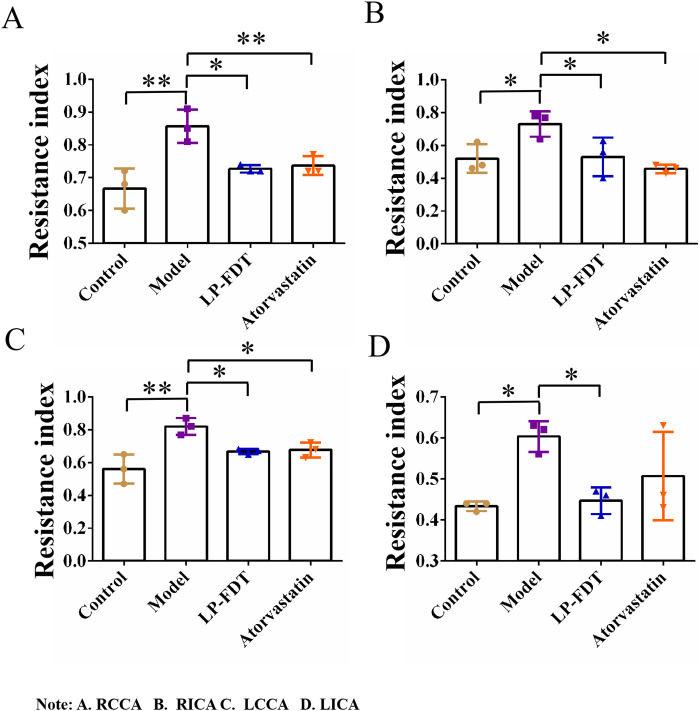
Vascular resistance index (RI) of four carotid arteries (A.RCCA, B.RICA, C.LCCA, D.LICA) detected by ultrasound.

To accurately predict the success rate of the animal models and the effects of drug interventions, multi-modal ultrasound was performed prior to the study endpoint. The results showed that the right common carotid artery (RCCA) and left common carotid artery (LCCA) of mice can be clearly detected by ultrasound. AS-plaques on the arterial walls of the mice were also successfully detected (Figure 2). With the contribution of ultrasound, the plaque detection rates in each group were 0/10 (0%) in the normal control group, 8/10 (80%) in the model group, 3/10 (30%) in the LP-FDT treatment group, and 2/10 (20%) in the atorvastatin treatment group (data not shown). Next, blood flow velocity was analyzed by specific ultrasonic module, and the resistance index (RI) of blood flow was calculated using the formula: RI=(PSV-EDV)/PSV (note: PSV: peak systolic velocity; EDV: end diastolic velocity). As shown in Figures 3, 4, the resistance index of blood flow in the RCCA, LCCA, RICA, and LICA was significantly higher in the model group compared to the normal control group (*P* < 0.01). Compared with the model group, LP-FDT treatment significantly reduced the resistance index of the RCCA and LICA (*P* < 0.05). Based on the ultrasound results, we accurately selected seven mice for follow-up experimental studies.

### 3.2 The LP-FDT treatment had no obvious effect on lowering blood lipids

**FIGURE 5:**
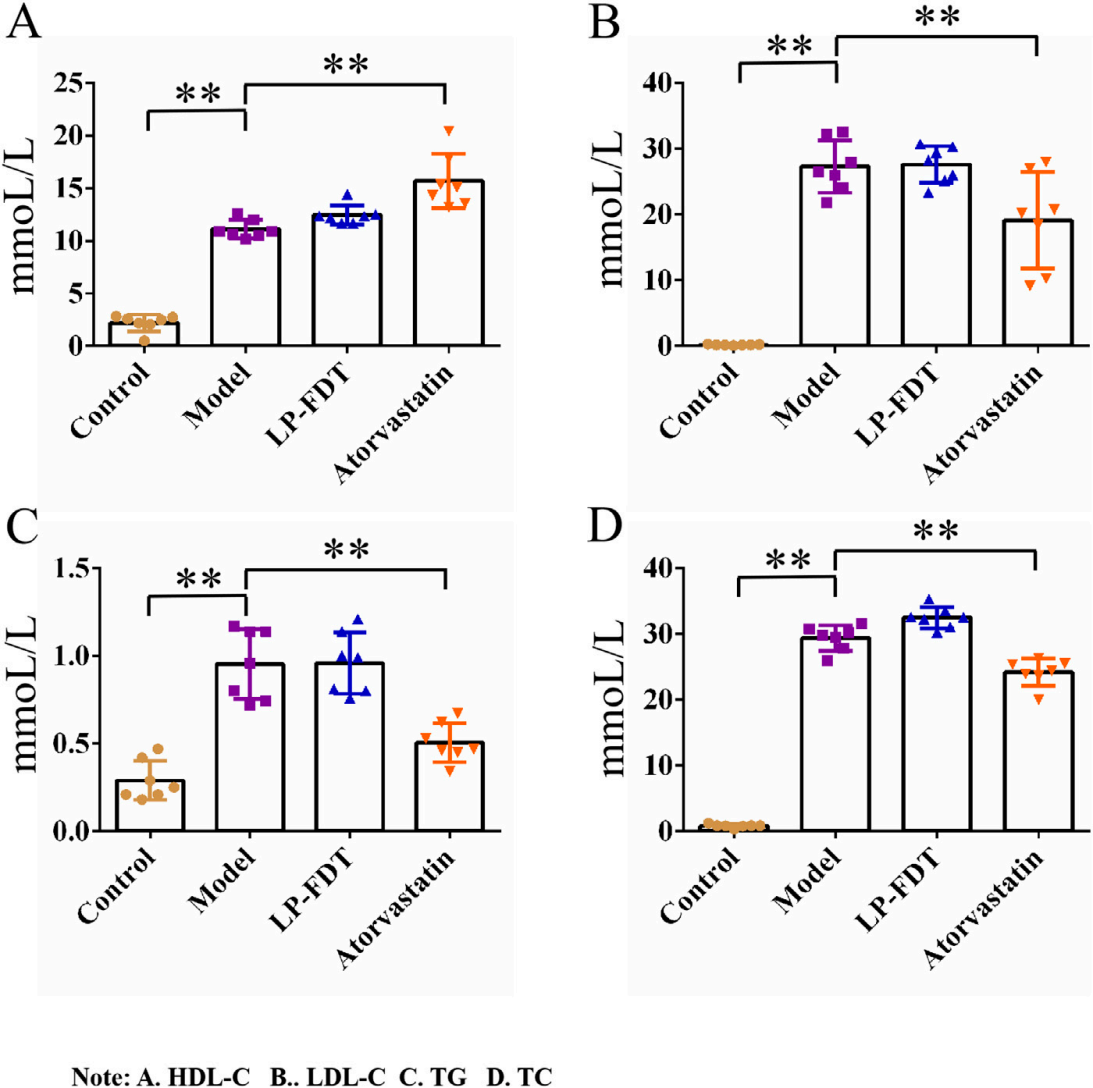
Serum biochemical analysis results (HDL-C, LDL-C, TG, TC). Note: **(A)** HDL-C, **(B)** LDL-C, **(C)** TG, **(D)** TC.

The results of the serum biochemical analysis showed that the high-fat diet significantly increased the serum levels of TC, TG, LDL-C, and HDL-C in the mice of the model group (*P* < 0.01). Compared to the model group, atorvastatin treatment decreased the serum levels of TC, TG, and LDL-C, but significantly increased the level of HDL-C (*P* < 0.01). Notably, LP-FDT treatment had no improvement effect on the levels of TC, TG, LDL-C, and HDL-C in serum (Figure 5). The results indicate that LP-FDT may not lipid-lowering effect directly.

### 3.3 The LP-FDT treatment alleviated AS-associated pathological degree

**FIGURE 6:**
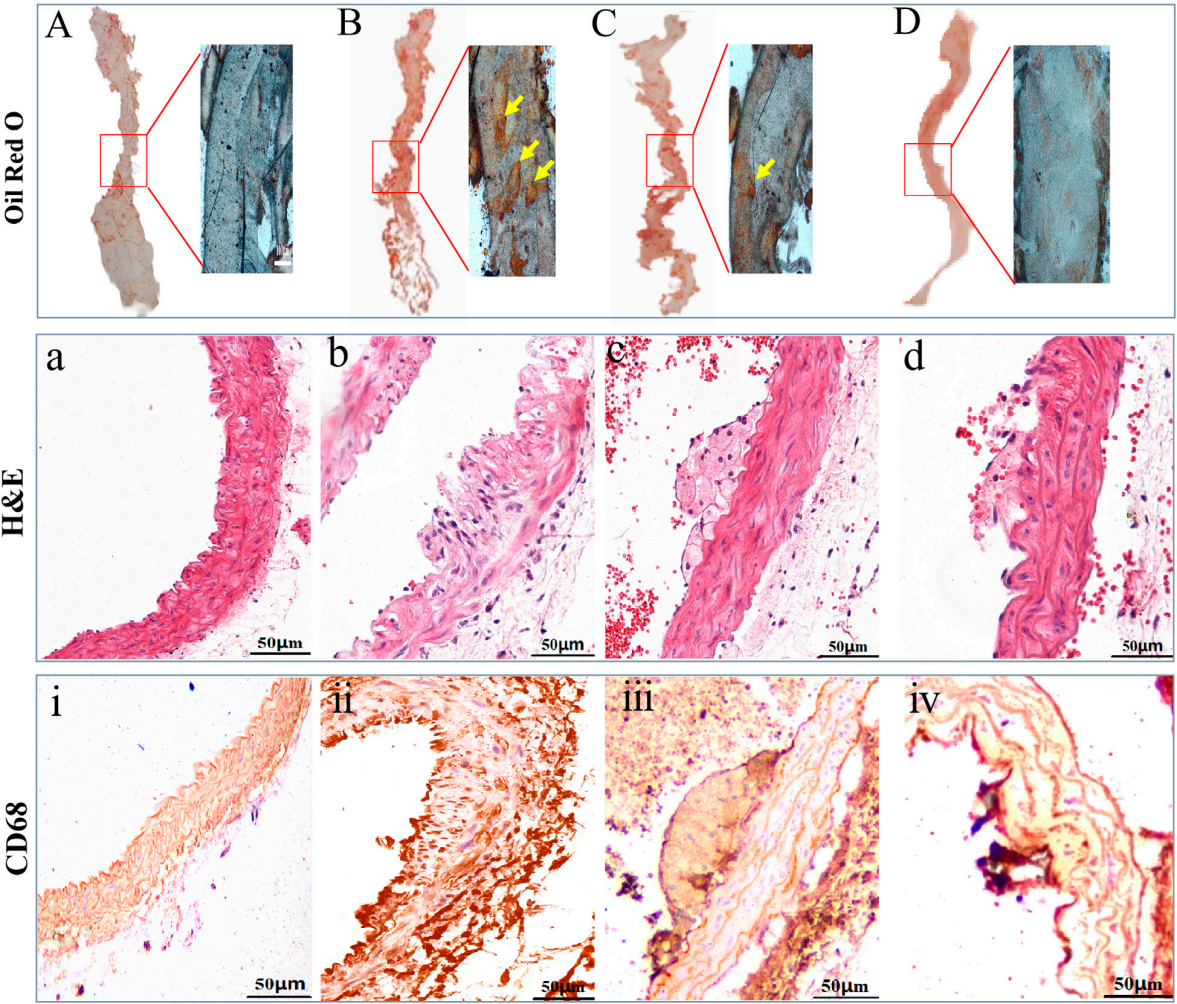
Results of oil red O, H&E, and IHC staining results of CD68. Note: (A), (a), and (ⅰ): normal control group; (B), (b) and (ⅱ): model group; (C), (c) and (ⅲ): LP-LDT treatment group; (D), (d) and (ⅳ): atorvastatin treatment groups.

H&E staining, Masson’s staining, and Oil red O staining were used to evaluate the pathological changes of the arterial wall tissue following LP-FDT treatment. As shown in Figure 6, dying with oil red O staining, a remarkably lipid deposition in the AS-plaque was detected on the aortic wall of model group mice (Figure 6B), which was significantly reduced in the LP-FDT and atorvastatin treatment groups (Figures 6C,D). H&E staining presented a typically form of AS-plaques in the model group mice. As showed in Figure 6b, the AS-plaque located in intima and extended into the media layer with a significant infiltration of inflammatory cells. Some plaques exhibited loss of the endothelial layer with significant fibrous cap rupture. Moreover, treatment with LP-FDT and atorvastatin dramatically reduce both the AS-plaque size, inflammatory cell infiltration, and improved the stability of the AS-plaque (Figures 6c,d). As indicated the macrophage in the lesion area of AS-plaque, CD68 positive expression was detected. As showed in Figure 6ⅱ, compared with normal control group, the macrophages in the AS-plaque increased significantly. However, treatment with LP-FDT and atorvastatin significantly reduce the CD68^+^ expression (Figure 6ⅲ,ⅳ).

### 3.4 The LP-FDT treatment reduced the expression of ECM in AS-plaque

**FIGURE 7:**
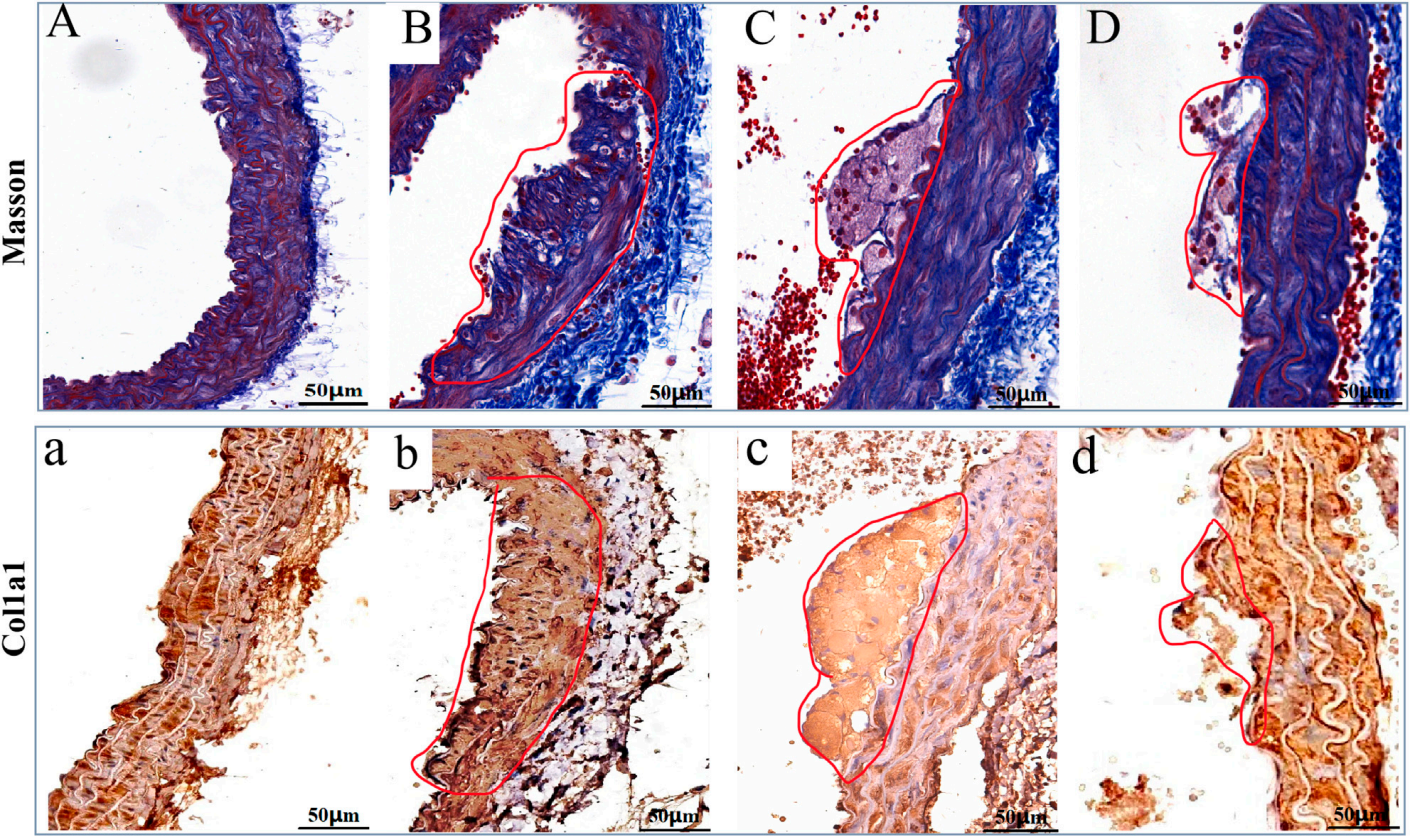
The Masson, and IHC staining results of COL1A1 on the AS-plaque in blood vessels. Note: (A) and (a) normal control group; (B) and (b) model group; (C) and (c) LP-LDT treatment group; (D) and (d) atorvastatin treatment groups.

As showed in Figure 7, Masson’s staining and IHC of COL1A1 highlighted the level of ECMs in the AS-plaque of the model group mice (Figures 7B,b), and the treatment of LP-FDT and atorvastatin significantly reduce the level of ECMs in the AS-plaque (Figures 7C,D,c,d). The results indicated that LP-FDT might play a role on the degradation of ECMs.

### 3.5 The LP-FDT treatment downregulated MMPs gene transcription and expression

Real time quantitative RT-PCR were employed to assess the gene transcription of MMPs, the results showed (data not show, see S2) that compared to the normal control group mice, the levels of all kinds of MMPs gene transcription were significantly increase in the AS model group mice. Amazingly, our data showed the level of MMPs gene transcription kept rising steadily on LP-FDT treatment group mice. And the degree of increase in gene transcription level in the LP-FDT treatment group was higher than that in the atorvastatin treatment group.

**FIGURE 8:**
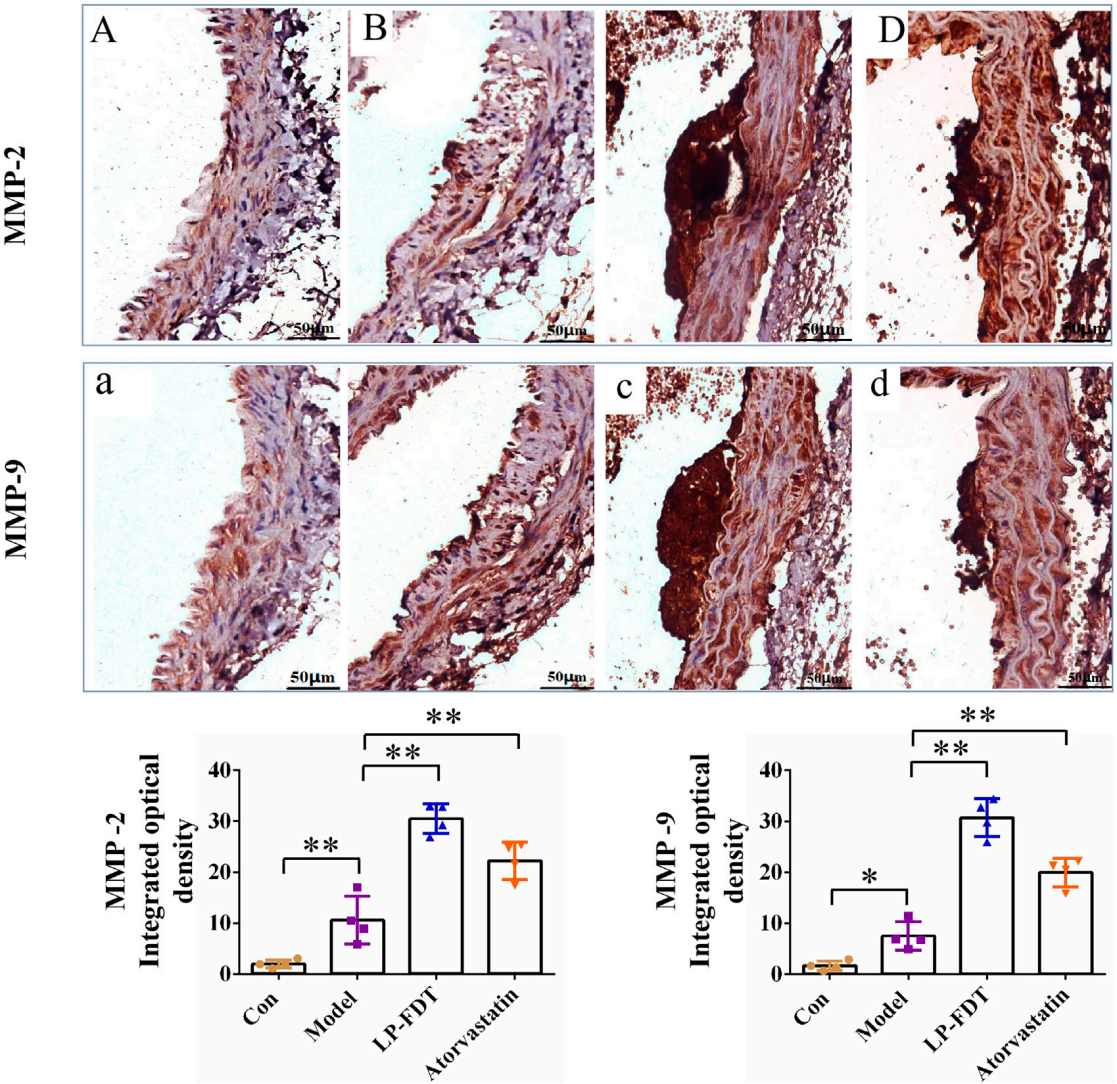
IHC staining results of MMP-2 and MMP-9 on the AS-plaque in blood vessels. Note: (A) and (a) normal control group; (B) and (b) model group; (C) and (c) LP-LDT treatment group; (D) and (d) atorvastatin treatment groups.

To verify the gene expression of MMPs, the protein expression of MMP-2 and MMP-9 was detected by IHC method, the results shown in Figure 8. Compared to the normal control group, the levels of MMP-2 and MMP-9 expression were significantly upregulated in the model group (*P* < 0.01). Notably, compared to the model group, the levels of MMP-2 and MMP-9 expression were continuously upregulated in both the LP-FDT and atorvastatin treatment group (*P* < 0.01). The seemingly contradictory data have posed certain challenges to the existing arguments.

## 4 Discussion

### 4.1 Vascular extracellular matrix is a potential target in the treatment of AS

Atherosclerosis (AS), a prevalent vascular disease, is a primary contributor to the staggering global burden of cardiovascular disease morbidity and mortality (Herrington et al., 2016; Barquera et al., 2015). It transcends age barriers, afflicting not only the middle-aged and elderly but is also increasingly observed in younger populations, thus emerging as a paramount challenge to global public health (Libby, 2021b). The pathogenesis of atherosclerosis is multifaceted, involving genetic predispositions, environmental factors, and complex interactions among various pathological mechanisms (Xu et al., 2018; Lechner et al., 2020; Ishida et al., 2024). The extracellular matrix (ECM) plays a crucial role in the pathogenesis of AS, contributing to endothelial dysfunction, lipid deposition, smooth muscle cell proliferation and migration, and plaque stability (Di Nubila et al., 2024; Gialeli et al., 2021). These biological processes are regulated by matrix metalloproteinases, such as MMP-2, and MMP-9. Some researchers believe that increased expression of MMP-2 and MMP-9 in AS-plaques is associated with a higher risk of rupture (Mohindra et al., 2021). Paradoxically, we study found that LP-LDT treatment upregulated both gene and protein expression of MMP-2 and MMP-9. A possible explanation for this observation is the activation of MMPs has a dual effect in atherosclerosis (Newby, 2005). It is generally believed that the activation of MMPs in the late stage of plaque will cause the instability and rupture of the plaque, but at the stage of plaque formation, MMPS can also have positive effects on atherosclerosis by degrading the ECM in the fibrous cap and dilating the blood vessels (Mason et al., 1999; Lemaître et al., 2001). Some studies have found that the expression of MMP-1 and MMP-2 is also increased and the ECM is decreased after the patients take statins (Ogita et al., 2013; Wu et al., 2012). The main drug component of LP-FDT is lumbricus protein, which can also activate MMP, so it may achieve the effect of prevention and treatment of AS by activating the expression of MMP series and degradation of ECM (Wang et al., 2016). From the pathological examination (Figure 6), the atherosclerotic plaques in APOE (−/−)+ mice induced by high-fat diet for 8 weeks in this study belong to the category of unstable plaques, and after LP-FDT treatment, the plaques are reduced and tend to be stable, so LP-FDT may be activated by MMP. Degradation of ECM to achieve plaque remodeling and reduction.

The main ingredients of LP-LDT, including peptide components, such as lumbrokinase, collagenase, and plasmin, may have indirect or direct effects on immune cells (Nakajima et al., 1993; Massicotte et al., 2003), indicating a multifaceted pharmacological action. According to some published studies, LP-FDT could disrupt the fibrous cap of atherosclerotic plaques, promote thrombolysis, prevent thrombosis, and improve the body’s microcirculatory function (Bäck et al., 2010; Keragala and Medcalf, 2021). Consequently, we suggest that LP-LDT leads to the secretion of high levels of MMPs, which in turn degrade fibrous tissue in plaques. Corresponding research is being carried out in our upcoming studies about how about how LP-FDT regulates MMPs.

### 4.2 Ultrasound technique in the context of experimental design for 3R principle

The 3Rs principles (reduction, replacement, and refinement) are central to humane experimental design. Since 1959, the principle of 3Rs have driven the development and use of methods that improve animal welfare by non-invasive techniques, such as micro-X-ray computed tomography (micro-CT), magnetic resonance imaging (MRI), and ultrasound. These imaging techniques, due to their repeatability, predictability, and non-invasiveness, significantly reduce the number of animals required for experimentation (Obrecht et al., 2023). For example, in the present study protocol, detecting statistically significant differences (*p* = 0.05) in AS-plaques would typically require group sizes of at least 44 mice based on the formula for sample size calculation: n = 2 (*Ζ*_*α*_+*Ζ*_*β*_)^2^×*σ* ^2^/Δ^2^ (note: n: Number of animals per group; *Ζ*_*α*_ *= 1.96*; *Ζ*_*β*_ = 0.84; *σ* = 5; Δ = 3). However, by using multi-mode ultrasound detection, we could predict the effective sample size and standard deviation based on plaque number and blood flow velocity in four arteries (*Ζ*_*α*_ *= 1.96*, *Ζ*_*β*_ = 0.84, *σ* = 3, Δ = 5). Therefore, a sample size of n = 6 mice was sufficient to demonstrate the pharmacological effects.

On the other hand, multi-mode ultrasound diagnosis at the 8th week confirmed the success of the AS model, eliminating the need to prolong the modeling and administration period, thus reducing the pain of the experimental animals. Considering this, the experimental research protocol was recognized and supported by the IACUC (Supporting Data 3).

### 4.3 LP-FDT is a potential innovative drug for preventing AS

The use of earthworm (*Lumbricus*) was first documented in *Shennong’s Classic of Materia Medica* and later classified as an insect-derived medicine in the *Compendium of Materia Medica* during the Ming dynasty. Modern pharmacological studies have demonstrated its anti-inflammatory, analgesic, calming, and thrombolytic properties. It is commonly applied in the treatment of chronic airway diseases, acute cerebral ischemia, epilepsy, and other conditions. To address the challenges of dysphagia and compliance, this study utilized lyophilization technology to develop a sublingual fast-dissolving tablet of lumbricus protein (LP-FDT). The main components of LP-FDT include pullulan (32.03%), mannitol (32.03%), earthworm protein (16.02%), saccharose (12.81%), and lemon extract (6.41%) (Supporting Data 1). This innovative dosage form ensures rapid disintegration, enabling prompt absorption and systemic distribution of the active ingredients. These characteristics allow for precise, targeted action and provide swift preventive and therapeutic effects. This work not only represents a significant extension of Traditional Chinese Medicine (TCM) theory but also exemplifies the seamless integration of TCM with Western medicine, combining traditional knowledge with modern technology. It paves the way for a novel approach for the prevention and treatment of AS.

## 5 Conclusion

In the present study, although the LP-FDT did not demonstrate a significant hypolipidemic effect, it showed notable efficacy in reducing AS-plaques in the AS model of high-fat diet-induced ApoE−/− mice. By employing multi-mode ultrasound, we aimed to reduce the number of animals used under the guidance of the 3Rs principles. The paradoxical results of the gene and protein expression of MMP-2 and MMP-9 have sparked new ideas for exploring the biological significance and molecular mechanisms behind the upregulation of MMP-2 and MMP-9 by LP-LDT.

## Data Availability

The original contributions presented in the study are included in the article/Supplementary Material, further inquiries can be directed to the corresponding authors.
