# Supplementary material for: Contribution of multimodal ultrasound in evaluating the efficacy of lumbricus protein fast dissolving tablets against atherosclerotic plaques in ApoE(−/−) mice
Source: Front Pharmacol. 2025 May 30;16:1551833. doi: 10.3389/fphar.2025.1551833 (PMC12163061; doi:10.3389/fphar.2025.1551833)

Supporting data 2. **Using Real time quantitative RT-PCR to examine the level of MMPs gene transcription**

1.The primer design of MMPs

GENE	FORWARD	REVERSE
MMP-1	ACAGTTGACAGGCTCCGAGAAATG	CCACATCAGGCACTCCACATCTTG
MMP-2	ACCATGCGGAAGCCAAGATGTG	AGGGTCCAGGTCAGGTGTGTAAC
MMP-3	TCGGGTTGGAGATGACAGGGAAG	TGAAGCCACCAACATCAGGAACAC
MMP-7	GAACACTCTAGGTCATGCCTTCGC	TCACTCCTGCGTCCTCACCATC
MMP-8	TGGGCTCTAAGTGGCTATGACCTG	ATCAATGGCTTGGACACTCCTTGG
MMP-9	CGCCACCACAGCCAACTATGAC	CTGCTTGCCCAGGAAGACGAAG
MMP-10	TCGGGTTGGAGATGACAGGGAAG	TGAAGCCACCAACATCAGGAACAC
MMP-11	CGAGGTGGAGACTATTGGCGTTTC	CAGCATCCTGGAAGGCAGCATC
MMP-12	AGCAACTGGGCAACTGGACAAC	CCGCTTCATCCATCTTGACCTCTG
MMP-13	ACAGTTGACAGGCTCCGAGAAATG	CCACATCAGGCACTCCACATCTTG
MMP-14	TCCGAGAAGTGCCCTATGCCTAC	GTGTACTGTCGCCGTGGAAACC
MMP-15	GGCAGATGGTGACAGCAAGGAAG	AATACAGAGCAACAGCAGCAAGGG
MMP-16	TTATGTGCTACAGTCTGCGGAACG	GGGGTCAGTCGGTGGAAGGTAG
MMP-17	TGGCGGATGCGGAGGATGTAG	GTGGAGTCAGAAGCAGCAGAGATG
MMP-19	TGGCTCCTGTCTATGCTGGCTAC	TCTCTTGTCTCTGGGCTCCTCTTG
MMP-20	TGCACTTGCCAACAGGGATTCG	CCTCGTTCAGCCACTTCGTAAGC
MMP-21	TAGCCCAAAGGAGTCAGCAGGAG	GCCAGTGTAGGTGAGTCCAATTCC
MMP-22	GGGCAGCTCAGGGAAATGTAGATG	CAGCGTGTAGCGGCGTCTTC
MMP-24	AGAAGGAGGTAGAGCGGCGTAAG	AGCAGCACCAGGAGGCAGAG
MMP-25	CCTGACCTCCTCCAGACTTCCAC	GGCAGCAGCGGAACCAACAG
MMP-27	CCCCAAATCCATCCACACACTCG	TCTGTCCATTGCTTGTGCCATCTC
MMP-28	CTGTAGATGGGCAATGGCGACTG	CTGTAGTGGACGAGGCTCTGAAAC
GAPDH	GGTGAAGGTCGGTGTGAACG	CTCGCTCCTGGAAGATGGTG

2.Experimental procedures

RT-PCR analysis was performed on a RT-PCR machine. All reactions were run in triplicate. The cycle threshold (Ct) method was used to calculate values. Ct values were normalized to the GAPDH gene as a cDNA loading control, and changes were calculated relative to controls. Total RNA was extracted from the vessel with a Trizol & Trizol PAL (CAT# R1200; LabLead) kit, and reverse transcription was subsequently performed with an All-in-One First-Strand Synthesis MasterMix kit (CAT# F0202; LabLead), A MagicSYBR Mixture kit (CAT# CW3008H; CWBIO) was used to quantify the mRNA levels of 22 MMPs.

3.Result of the level of MMPs gene transcription

The qPCR results of 22 MMPmRNA in mouse aorta showed that the MMP in the Model group was higher than that in the Con group, which may be related to the instability of plaque. The mRNA of each MMP detected in the LP-FDT group was significantly higher than that in the Model group. Most MMP in the Atorvastatin group was higher than that in the Model group.The results suggest that LP-FDT may inhibit and reduce the ECM in plaque by regulating the expression of MMP series genes.

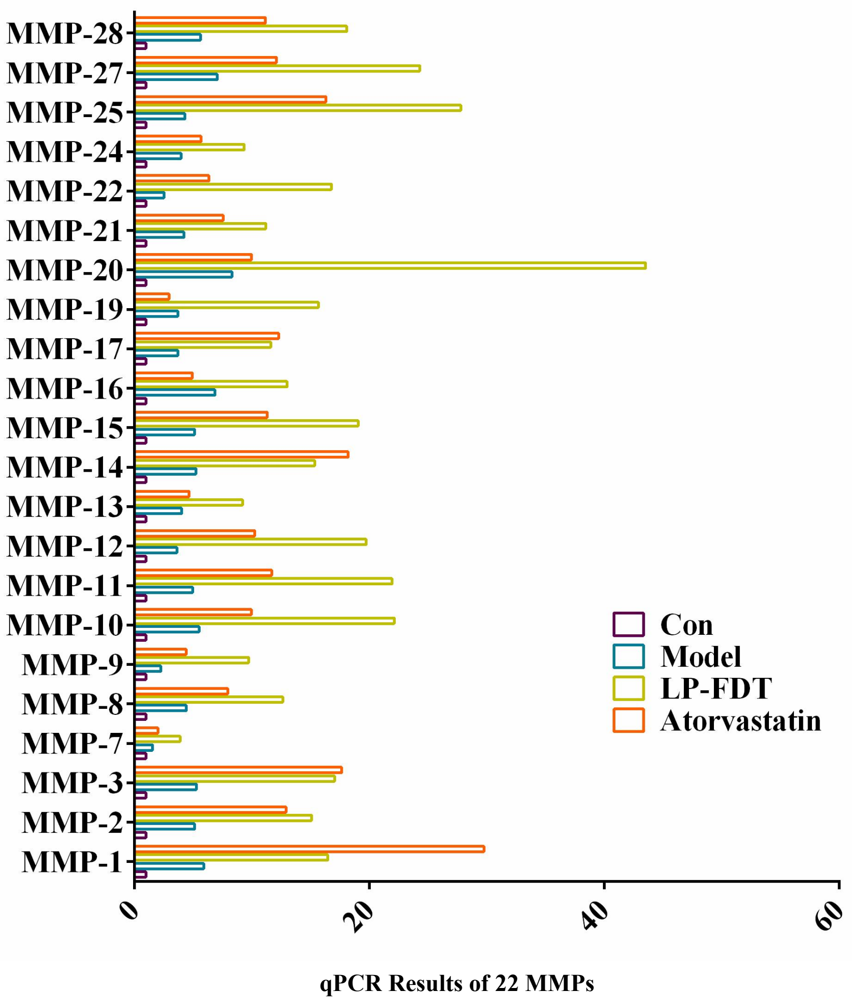

Supplement: Supplementary file 1 [file DataSheet2.pdf]
