# Supplementary material for: Contribution of multimodal ultrasound in evaluating the efficacy of lumbricus protein fast dissolving tablets against atherosclerotic plaques in ApoE(−/−) mice
Source: Front Pharmacol. 2025 May 30;16:1551833. doi: 10.3389/fphar.2025.1551833 (PMC12163061; doi:10.3389/fphar.2025.1551833)
Supplement: Supplementary file 2 [file DataSheet3.pdf]

Beijing University of Chinese Medicine animal experimental ethics review form

ID(Nọ): BUCM-2024022710-1094

<table>
<tr><td rowspan="6">The relevant information filled in by the applicant</td><td colspan="2">applicant: Zhihao Lu</td><td colspan="2">Laboratory animal registration number: 1121043000262</td></tr>
<tr><td colspan="4">Experiment Name: Observation of curative effect of fast dissolving tablets of lumbricus protein (LP-FDT) in preventing and treating atherosclerosis model mice</td></tr>
<tr><td rowspan="3">Animal condition</td><td colspan="3">Animal source:Beijing Charles River Experimental Animal Technology Co., LTD</td></tr>
<tr><td colspan="3">Varieties and strains: APOE-/-; C57BL/6J Level: SPF</td></tr>
<tr><td colspan="2">quantity: 45</td><td>Application date: Feb 27, 2024</td></tr>
<tr><td colspan="4">Key points of the experiment: (including disease modeling, surgical methods, methods of killing animals after the experiment, etc.)
1.Modeling and surgical methods:
The mice were fed high-fat diet for 8 weeks and were given sublingual intravenous medication every day. Material collection on the 8th weekend.
2.Execution of experimental animals and disposal of corpses:
Execution: Spinal dislocation method
Disposal of mouse carcasses: euthanized animals will be released to designated locations in the animal room.

Applicant's signature: Zhihao Lu Contact number: 13685336755</td></tr>
<tr><td>Project leader commitment</td><td colspan="4">The above contents are true, and I take full responsibility for the scientific, rational and feasible design of animal experiments.
Signature of project leader: Jian Li Date: Feb 27, 2024</td></tr>
<tr><td>Review basis</td><td colspan="4">1. Does the project have to use experimental animals for experiments, that is, can non-living methods such as computer simulation and cell culture be used instead of animals or lower animals instead of higher animals for experiments?
2. Are the applicant's qualifications and the varieties, quality grades and specifications of the animals used appropriate? Can the number of animals used be reduced by improving the design scheme or using high-quality animals?
3. Can we optimize the experimental program and treat animals well by improving the experimental method, adjusting the experimental observation index and improving the method of killing animals?</td></tr>
<tr><td>Review result Do you agree or disagree</td><td colspan="4">Audit comments of the implementing unit:
Signature: 赵保胜印 Feb 27, 2024</td></tr>
</table>

CS 扫描全能王
3亿人都在用的扫描App

Applicant's Experimental scheme)	Comments of the Experimental Animal Ethics Subcommittee of the Academic Committee of Beijing University of Chinese Medicine:<br>北京中医药大学 医学与实验动物伦理委员会 1100000013479<br>(Signature) 王停之印<br>Feb 27, 2024

CS 扫描全能王
3亿人都在用的扫描App
