# Supplementary material for: Contribution of multimodal ultrasound in evaluating the efficacy of lumbricus protein fast dissolving tablets against atherosclerotic plaques in ApoE(−/−) mice
Source: Front Pharmacol. 2025 May 30;16:1551833. doi: 10.3389/fphar.2025.1551833 (PMC12163061; doi:10.3389/fphar.2025.1551833)
Supplement: Supplementary file 3 [file DataSheet1.pdf]

Supporting data 1. The characteristics of the fast-dissolving tablet of lumbricus protein (LP-FDT)

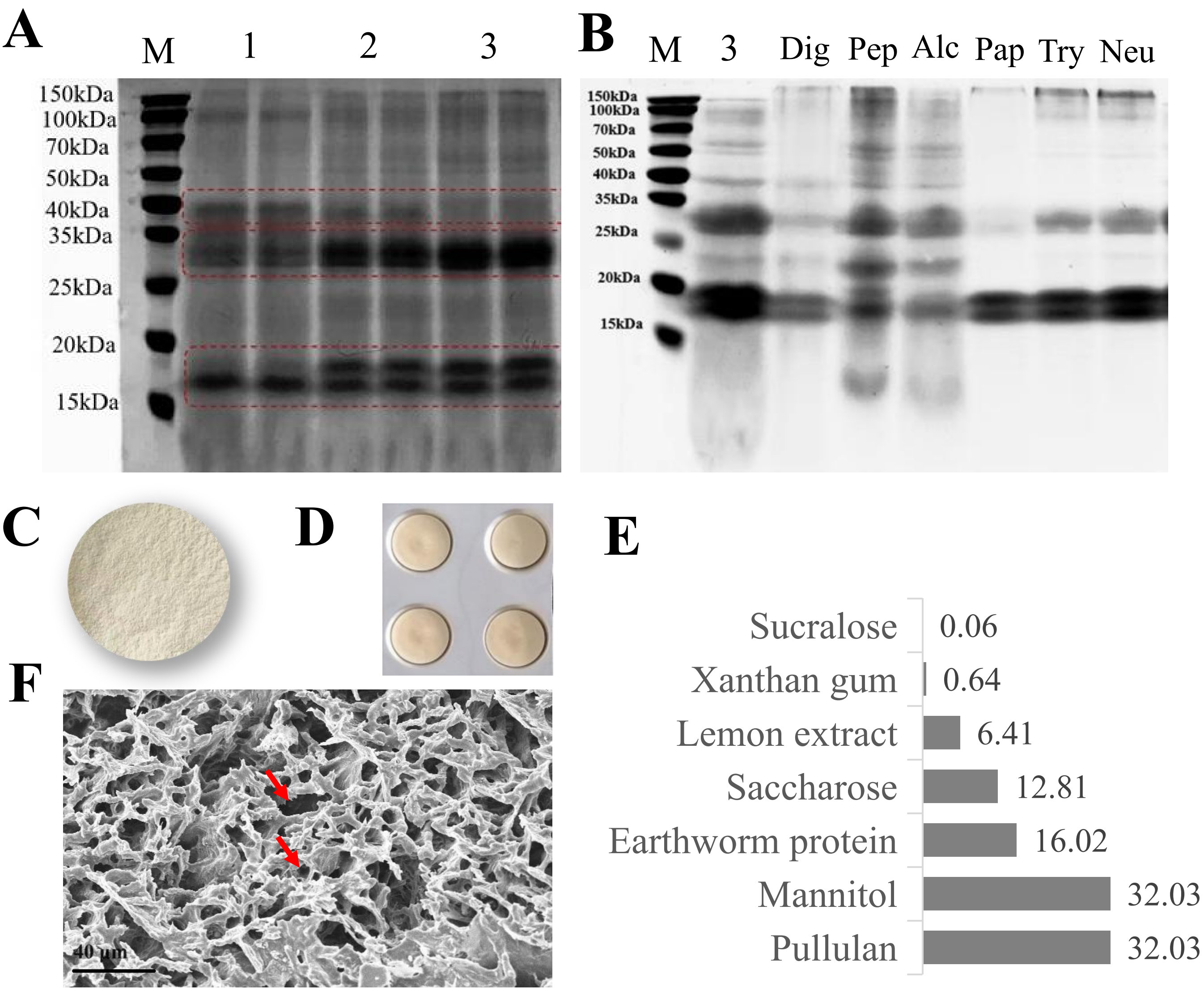

Note:

A. SDS-PAGE of lumbricus protein;

B. SDS-PAGE of different enzymatic hydrolysis products;

C. appearance of LP-FDT;

D. Mold appearance for preparing LP-FDT;

E. the composition of the LP-FDT;

F. the SEM image of LP-FDT
